# Brief report: Meth, tranq, gas station heroin & other drugs complicating addiction treatment

**DOI:** 10.1111/ajad.70151

**Published:** 2026-02-25

**Authors:** Erin L. Winstanley, Jane M. Liebschutz, Cristina Murray‐Krezan, Galen E. Switzer, Samantha Nash, Sarah Kawasaki

**Affiliations:** ^1^ Division of General Internal Medicine, Center for Research on Health Care, School of Medicine University of Pittsburgh Pittsburgh Pennsylvania USA; ^2^ UPMC Pittsburgh Pennsylvania USA; ^3^ Division of General Internal Medicine, Center for Biostatistics and Qualitative Methodology, School of Medicine University of Pittsburgh Pittsburgh Pennsylvania USA; ^4^ Department of Psychiatry and Behavioral Health and Internal Medicine Penn State Milton S. Hershey Medical Center Hershey Pennsylvania USA

## Abstract

**Background and Objectives:**

Patients using fentanyl have worse treatment outcomes; however, little is known about other drugs that complicate treatment.

**Methods:**

A national survey (*n* = 396) was conducted using a random sample of clinicians waivered to prescribe buprenorphine in the United States. This study reports the results of a single survey item on clinicians' perceptions of other drugs, besides IMF, complicating treatment.

**Results:**

Clinicians reported methamphetamine (86.4%), synthetic cannabinoids (42.7%), and xylazine (41.4%) were complicating treatment; reports varied by geographic region.

**Conclusions and Scientific Significance:**

Rapid clinician surveys can provide real‐time data on changing patterns of drug use's impact treatment outcomes.

## INTRODUCTION

In recent years polysubstance use has become increasingly complicated due to higher drug potency, dynamic drug markets and emerging novel psychoactive substances (NPS). Co‐use of substances may be intentional (e.g., to potentiate or counteract a drug effect) or unintentional (e. g., adulterants or containments)^1^; either way we often know little about the pharmacology of NPS^2^ and perhaps even less about how concurrent use impacts the risk of overdose or treatment outcomes. Near real‐time national data on NPS use are scarce and often initial reports are described in the media or in a case report(s). The media initially described the emergence of new drugs, like xylazine (“tranq”) and tianeptine (“gas station heroin”), which make it difficult to estimate the prevalence of use or ascertain whether use is occurring in isolated geographic areas. Illicitly manufactured fentanyl (IMF), which is now largely ubiquitous across the United States (US), has been associated with a higher risk of overdose death due to its potency and worse treatment outcomes.^3^ More specifically, IMF has been associated with precipitated opioid withdrawal when initiating buprenorphine treatment and patients using IMF have lower rates of retention in treatment and higher rates of relapse to illicit drug use.^3^ Polysubstance use has also been associated with higher rates of relapse.^4^

The purpose of this brief report is to identify drugs, other than IMF, that clinicians report as complicating addiction treatment initiation for patients with opioid use disorder (OUD). Challenges initiating treatment for patients with OUD onto buprenorphine have largely been attributed to IMF; however, it is highly probable that other drugs are also complicating treatment given the dynamic drug supply and patterns of concurrent drug use that includes NPS. Epidemiological surveillance data provide insights on drugs present in overdose fatalities, but data lags cause delays in identification of changing patterns of drug use and these data provide little insight on how treatment outcomes may be impacted.

## METHODS

A web‐based survey conducted from June 2023 to March 2024 was distributed to a stratified random sample of buprenorphine waivered clinicians in the United States. The sampling frame was the US Drug Enforcement Administration (DEA) registrant database. The strata included prescriber type, number of patients waivered to treat, and census region. The survey purpose was to identify clinician‐reported problems when initiating buprenorphine treatment among patients using fentanyl. To be eligible, clinicians reported inducting >9 patients onto buprenorphine in the past year and having done ≥1 buprenorphine inductions in the past 90 days. More details regarding the survey instrument and methods are available elsewhere.^5^ This analysis includes results from a single survey item, “Besides fentanyl and fentanyl analogs, which other drugs are complicating treatment for patients with opioid use disorder?” Response options included: methamphetamine or other illicit psychostimulants, xylazine, novel opioids (e.g., etonitazene and analogs), designer or non‐FDA approved benzodiazepines (e.g., etizolam and analogs), synthetic cannabinoids (e.g., K2/spice), synthetic cathinones (e.g., bath salts), tianeptine (“gas station heroin”), or other. If ‘other’ was selected, participants were able to specify the other substance name in an open‐text field. The survey response rate was 26.1% and participants who completed 50% of the survey items were excluded (*n* = 9) per the protocol. Data results are presented as frequency distributions and are also aggregated by the jurisdiction (states + Washington DC) in which the clinician provided clinical care.

## RESULTS

Participants (*n* = 396) represented 47 states and the District of Columbia (48 jurisdictions).

46.8% were male, 77.9% were White, 6.7% were Hispanic, and the mean age was 47 (SD = 12.1). The majority of participants were physicians (60.7%) or nurse practitioners (30.2%). Participants worked in a variety of clinical settings and the primary setting was reported most frequently as a primary care practice (36.9%), opioid treatment program (19.0%), or outpatient community mental health center (14.0%).

**Figure 1:**
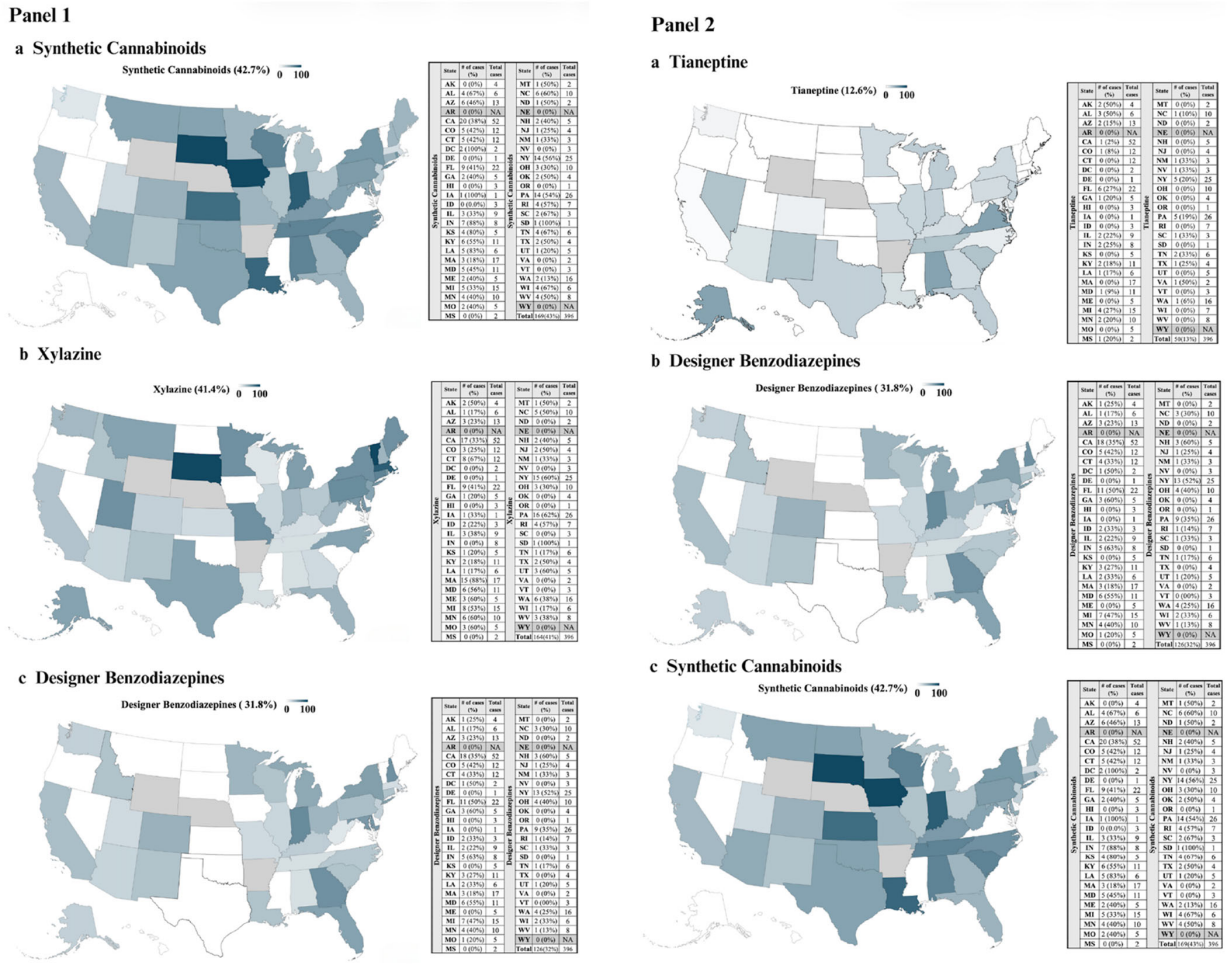
Number of clinicians reporting specific drugs that complicate treatment by state.

Most clinicians (86.4%) reported methamphetamine or other illicit psychostimulants as complicating treatment which was followed by synthetic cannabinoids (42.7%), xylazine (41.4%), designer benzodiazepines (31.8%), and synthetic cathinones (15.4%). More than 10% endorsed complications from tianeptine (12.6%) and novel opioids (12.5%). Several clinicians specified an “other” drug(s) (*n* = 46). In the open text field clinicians were able to specify more than one drug, and the most frequently written responses were kratom (*n* = 15), benzodiazepines (*n* = 12), and cocaine (*n* = 9). Figure 1 displays the states with clinicians reporting the specific drugs (Panel A: novel opioids, xylazine, synthetic cathinones, and Panel B: tianeptine, designer benzodiazepines, and synthetic cannabinoids) that were complicating treatment and an adjacent table that displays the proportion of clinicians within that state. Clinicians, across nearly all regions of the United States, reported that these drugs were complicating treatment.

## DISCUSSION

The drug category “methamphetamine or other illicit psychostimulants” was most frequently endorsed by clinicians as complicating treatment, which is consistent with previous reports that methamphetamine use has become ubiquitous across the United States.^6^ While xylazine use has been reported to be more common in the Northeastern/Mid‐Atlantic regions of the United States,^7^ xylazine has been detected in urine toxicology from 64% (25/39) of states.^8^ Our study found that clinicians in 77% (37/48) of jurisdictions reported xylazine as complicating treatment. The FDA has issued several warnings regarding tianeptine, and calls to national poison control centers for tianeptine exposures increased significantly 2013–2017.^9^ While few clinicians reported tianeptine as complicating treatment, these clinicians represented about half of jurisdictions (25/48), which was surprising given that a recent systematic review found only 52 cases of tianeptine misuse described in the published literature.^10^

Substances like synthetic cannabinoids and cathinones, as well as tianeptine, may be widely available across the US if they are unscheduled and labeled “not for human consumption.”^2^ Unlike scheduled drugs, their pharmacological characteristics are often unknown and/or there is insufficient empirical evidence of abuse potential or negative health effects. However, as an unregulated drug market, there is no oversight of ingredients or potency. In these instances, clinician reports of how these substances are complicating treatment may provide early insights that could inform the need for targeted epidemiological surveillance or the need to rapidly conduct empirical clinical research.

There are several limitations of this study. First, the survey reports on clinicians' subjective reports of drugs complicating OUD treatment rather than objective patient‐level toxicological data. While standard toxicological testing used in clinical settings may include drugs like methamphetamine and fentanyl, rarely is comprehensive NPS testing available even through an external laboratory. The survey included a relatively limited list of NPSs and notably, it did not include medetomidine which has more recently emerged in overdose reports. The survey included a stratified randomized sample of clinicians prescribing buprenorphine; however, it was not powered to detect state‐level differences. Finally, clinicians who have experienced problems initiating buprenorphine in patients may have been more inclined to complete the survey, resulting in response bias.

In a national sample of clinicians who treat patients with OUD, the survey found that clinicians believe several other drugs besides IMF are complicating treatment. While there is existing research on the health risks of methamphetamine use and growing body of research on xylazine, less is known about how tianeptine and synthetic cathinones or cannabinoids may impact treatment outcomes. Additional research is warranted to (1) confirm patient use of these drugs using toxicological measurement and (2) understand how confirmed drugs are complicating treatment and to identify strategies to improve medical management. Given the lack of real‐time data on NPSs and their impact on treatment, rapid brief surveys of clinicians may help contextualize NPS surveillance data and understand medical consequences.

## CONFLICT OF INTEREST STATEMENT

The authors declare that there are no conflict of interests.

## References

[1] Ciccarone D . The Donovan Memorial Lecture: ‘Fentanyl‐Plus’: a new era of fentanyl polydrug combinations in the North American overdose crisis. J Med Toxicol. 2025;22:49‐62. 10.1007/s13181-025-01104-6 41214415 PMC12834897

[2] Baumann MH , Volkow ND . Abuse of new psychoactive substances: threats and solutions. Neuropsychopharmacology. 2016;41(3):663‐665. 10.1038/npp.2015.260 26303285 PMC4707839

[3] Volkow ND . The epidemic of fentanyl misuse and overdoses: challenges and strategies. World Psychiatry. 2021;20(2):195‐196. 10.1002/wps.20846 34002497 PMC8129846

[4] Pan Y , Feaster DJ , Odom G , et al. Specific polysubstance use patterns predict relapse among patients entering opioid use disorder treatment. Drug Alcohol Depend Rep. 2022;5:100128. 10.1016/j.dadr.2022.100128 36644227 PMC9838120

[5] Kawasaki SS , Liebschutz JM , Murray‐Krezan C , et al. Barriers to buprenorphine initiation in patients using fentanyl. JAMA Netw Open. 2026;9(1):e2552136. 10.1001/jamanetworkopen.2025.52136 41490107 PMC12771233

[6] Jones CM , Houry D , Han B , Baldwin G , Vivolo‐Kantor A , Compton WM . Methamphetamine use in the United States: epidemiological update and implications for prevention, treatment, and harm reduction. Ann NY Acad Sci. 2022;1508(1):3‐22. 34561865 10.1111/nyas.14688 PMC9097961

[7] Kariisa M , O'Donnell J , Kumar S , Mattson CL , Goldberger BA . Illicitly manufactured fentanyl‐involved overdose deaths with detected xylazine—United States, January 2019‐June 2022. MMWR Morb Mortal Wkly Rep. 2023;72(26):721‐727. 37384558 10.15585/mmwr.mm7226a4 PMC10328484

[8] Holt AC , Schwope DM , Le K , Schrecker JP , Heltsley R . Widespread distribution of xylazine detected throughout the United States in healthcare patient samples. J Addict Med. 2023;17(4):468‐470. 10.1097/ADM.0000000000001132 37579111 PMC10417214

[9] El Zahran T , Schier J , Glidden E , et al. Characteristics of tianeptine exposures reported to the National Poison Data System—United States, 2000‐2017. MMWR Morb Mortal Wkly Rep. 2018;67(30):815‐818. 30070980 10.15585/mmwr.mm6730a2 PMC6072055

[10] Parnia S , Jain L , Ali M , et al. Gas station heroin‐tianeptine and its impact: a systematic review and exploratory analysis. BMC Public Health. 2025;Oct 24 25(1):3591. 10.1186/s12889-025-24666-0 41136982 PMC12551324

